# Hypermetropic Headaches

**Published:** 1892-07-09

**Authors:** 


### OPHTHALMOLOGY.

### HYPERMETROPIC HEADACHES.

A large number of patients consult the oculist on account of headache, which has failed to be cured with medicaments, and which, though relieved by rest from their duties, returns again after any effort thrown on the visual powers. In a large number of these cases the fault lies in the eye being hyperopic, or hyperopic and astigmatic.

The hyperopic eye is, in ordinary civilised life, continually at work focussing images on the retina by means of increasing and diminishing the convexity of the crystalline lens, for as every object falls within its far point, which far point is infinity, it remains blurred unless an effort at accommodation be made. The closer the object is to the eye, the greater must be that effort at accommodation, to obtain a clear image, and likewise the smaller the object to be focussed the greater the exertion. Hence it is that patients with hypermetropic headaches belong to the classes who are constantly employed using their near vision, such as school children, sempstresses, clerks, watch makers, paper folders, boot finishers, students, and the like. These patients generally complain of a weight over the eyes, accompanied by a tight feeling, after prolonged application to their work, more especially occurring when working in an insufficiently lighted room or by artificial light; this passes off with rest, but recurs at shorter intervals on return to work, and eventually becomes a dull, aching pain, not situated in the eye itself, but over and around the orbit.

This headache does not occur in all hypermetropic persons, and often the subject goes on working for years before being troubled with headache, although hypermetropic all the while, the onset of the symptoms often commencing after ill-health, or excessive bodily fatigue, insufficient rest or food, from loss of appetite, or want of means for obtaining it. Then the patient finds that the words, or stitches, or other objects he is working at become blurred, or run into one another, or a cloud or mist comes between the object and himself, and if he still struggle on against these symptoms, he gradually finds his far vision also fail.

Now, how is one to diagnose this class of visual headache! The vision may be tested, and the result be $V = \frac{6}{6}$ and $J \frac{1}{6 \cdot 1_4}$ in both eyes, which means he can see normally both for near and far vision, but still he might not see the small print after prolonged attempts to read it. We must therefore discover if he can see as well through a convex glass, and the highest glass that he can see $\frac{6}{6}$ with is the glass that indicates the amount of manifest hypermetropia he possesses. This may be only one dioptre or several dioptres, for the headaches occur in those with hypermetropia of low degree, though more often in those with hypermetropia of high degree, and the glass that shows this will aid his near vision to a great extent. There is, however, latent hypermetropia also to be taken into account; thus after paralysing accommodation with atropine, he will probably require a stronger convex glass wherewith to see $\frac{6}{6}$ than he did before using the atropine, and the difference between these two glasses will be the amount of latent hypermetropia.

In cases where the distant vision has also become obscured by spasm of the muscle of accommodation, the result of vision testing may read $V \frac{6}{24}$ $J \frac{4}{6}{}_{12}$, which may make the eye appear myopic at first sight, but then such an eye if myopic would probably see $J \frac{1}{6}$. Paralysing the accommodation, or retinoscopy, will at once clear up this doubt.

Having decided that the headaches are due to hypermetropia, the treatment consists in perfect rest to the eyes, not only by cessation from work, but by paralysing the accommodation with atropine, and thus preventing also all involuntary action of the muscles of accommodation. In a person under the age of thirty it will be found necessary to keep up the effect of atropine for from a week to ten days, during which time the refraction should be carefully made out by direct ophthalmoscopy or by retinoscopy, and in another ten days, when the effects of the atropine have passed off, the patient should be fitted out with appropriate glasses, which will enable him to return to his ordinary duties without the constant recurrence of the headache.

